# Experience improves the performance of endobronchial ultrasound-guided transbronchial biopsy for peripheral pulmonary lesions: A learning curve at a medical centre

**DOI:** 10.1371/journal.pone.0179719

**Published:** 2017-06-20

**Authors:** Chun-Ta Huang, Sheng-Yuan Ruan, Yi-Ju Tsai, Chao-Chi Ho, Chong-Jen Yu

**Affiliations:** 1 Department of Internal Medicine, National Taiwan University Hospital, Taipei, Taiwan; 2 Department of Traumatology, National Taiwan University Hospital, Taipei, Taiwan; 3 Graduate Institute of Clinical Medicine, College of Medicine, National Taiwan University, Taipei, Taiwan; 4 Graduate Institute of Biomedical and Pharmaceutical Science, College of Medicine, Fu Jen Catholic University, New Taipei City, Taiwan; Postgraduate Institute of Medical Education and Research, INDIA

## Abstract

**Background:**

Endobronchial ultrasound(EBUS)-guided transbronchial biopsy(TBB) is the preferred diagnostic tool for peripheral pulmonary lesions(PPLs) and mastering this procedure is an important task in the training of chest physicians. Little has been published about the learning experience of physicians with this technique, particularly at an institutional level. We aimed to establish a learning curve for EBUS-guided TBB for PPLs at a medical center.

**Methods:**

Between 2008 and 2015, consecutive patients with PPLs referred for EBUS-guided TBB at National Taiwan University Hospital were enrolled. To build the learning curve, the diagnostic yield of TBB (plus brushings and washings) was calculated and compared. Meanwhile, lesion characteristics, and procedure-related features and complications were obtained to analyze associations with TBB yield and safety profile.

**Results:**

A total of 2144 patients were included and EBUS-guided TBB was diagnostic for 1547(72%). The TBB yield was 64% in 2008 and reached a plateau of 72% after 2010. It took approximately 400 EBUS-guided procedures to achieve stable proficiency. Further analysis showed that improvement in diagnostic yield over time was mainly observed in PPLs, in cases in which the diameter was ≤2 cm or the EBUS probe could not be positioned within. Complication rates were low, with 1.8% and 0.5% for pneumothorax and hemorrhage, respectively.

**Conclusions:**

Even though EBUS-guided TBB is an easy-to-learn technique, it takes 3 years or around 400 procedures for a medical center to achieve a better and stable performance. In particular, the diagnostic yield for lesions without the probe within or those sized ≤2 cm could improve with time.

## Introduction

Diagnosis of peripheral pulmonary lesions (PPLs) can be achieved with a variety of modalities, including bronchoscopy, video-assisted thoracoscopic surgery (VATS) and computed tomography (CT)-guided biopsy. Recent modifications of bronchoscopy with ancillary techniques, such as endobronchial ultrasound (EBUS), electromagnetic navigation and thin bronchoscopes,[1–6] have safely and significantly increased the diagnostic yield of bronchoscopy for PPLs. Among these, EBUS has gained the most widespread application, as a result of a barrage of evidence supporting its advantages in this field.[7, 8] More recent guidelines also recommend the use of EBUS-guided transbronchial biopsy (TBB) as the first diagnostic tool for sampling PPLs.[9–11] As such, it is clear that mastering this procedure is one of the most important tasks in the training of chest physicians.

A learning curve is a concept used to measure how soon one can be proficient in a skill. Understanding a single institutional or individual learning curve is of importance for setting standards, designing training programs and establishing competitive strategies. However, to the best of our knowledge, there is a paucity of such information about EBUS-guided TBB. Based on expert opinions, the guidelines recommend that each learner performs a minimum number of 40–50 EBUS-guided procedures for each learner to perform in order to achieve basic competence.[12, 13] In real-world practice, success in a bronchoscopic intervention is always based on teamwork; thus, it would be more practical to know about the learning curve at an institutional level.

Accordingly, in this study, we aimed to establish the learning curve for EBUS-guided TBB for PPLs at a medical centre. At the same time, we sought to investigate whether the changes in EBUS performance over time were similar with patients with different clinical features.

## Methods

### Study design and settings

This retrospective study was conducted in accordance with the amended Declaration of Helsinki at National Taiwan University Hospital, a medical centre in northern Taiwan. We implemented the use of EBUS-guided TBB for PPLs in 2007, and beginning in late 2007, patients with PPLs no longer underwent conventional TBB. Following approval from the Research Ethics Committee of National Taiwan University Hospital, consecutive patients with PPLs referred for EBUS-guided TBB between January 2008 and December 2015 were enrolled in this study. In our institution, the minimum diameter of the PPLs considered for EBUS-guided TBB was 8 mm. A PPL was defined as a lesion that was surrounded by lung parenchyma and was not seen within the bronchial trees during conventional bronchoscopy. Written informed consent was obtained from all patients before the bronchoscopic procedure.

### EBUS-guided TBB

The procedure was performed by chest fellows under the supervision of experienced pulmonologists. Conventional bronchoscopy with a 2.0-mm working channel (BF-P260F bronchoscope; Olympus, Tokyo, Japan) was first conducted to inspect the bronchial trees, after local anaesthesia with 5 ml of 2% lidocaine sprayed or nebulized into the upper airway mucosa and intramuscular injection of meperidine 50 mg if not contraindicated.[14, 15] Additional 1–2 ml of 2% lidocaine were instilled onto the larynx, carina and second carina following insertion of the bronchoscope. EBUS was then performed with an endoscopic ultrasound centre (EU-M30S; Olympus) and a 20-MHz radial-type ultrasonic probe (UM-S20-20R; Olympus). After locating the PPLs on the EBUS images, EBUS-guided TBB was performed as described previously.[16] The biopsy procedures were repeated until at least 2 adequate samples were retrieved. Bronchial washing or brushing was conducted after TBB, based on the judgment of the pulmonologists in charge. Other ancillary bronchoscopic techniques, including guiding sheath, ultrathin bronchoscopy, fluoroscopy, virtual bronchoscopic navigation, electromagnetic navigation, and rapid on-site evaluation, were not applied in this study.

### Outcomes of interest

The primary outcome of interest was the diagnostic yield of EBUS-guided TBB of PPLs across the study period. The TBB was also considered diagnostic if the diagnosis of a PPL was achieved via bronchial washing or brushing during the same bronchoscopy session. As for other non-diagnostic PPLs, the final diagnosis was established by pathologic evidence from CT-guided or VATS biopsy, or other invasive diagnostic procedures, microbiologic analysis, serology testing or clinical follow-up. Other outcomes of interest included the time-varying changes in the diagnostic yield of EBUS-guided TBB of lesions with distinctive features. These features were pre-defined as those patient characteristics associated with a diagnostic EBUS-guided TBB of PPLs.

### Clinical information

Medical records were reviewed in detail for demographic data, lesion size, location and appearance on CT, ultrasonic probe position on EBUS images, final diagnosis, and bronchoscopy-associated complications. Lesion size was measured as the greatest axial diameter on CT scans. Lesion location was categorized into 5 anatomic lobes. If the PPL resided in 2 lobes of the lung, it was assigned to the lobe containing more than half of the lesion area. The lesion appearance was characterized as a solid, partly solid, pure ground-glass or cavitary opacity. The position of the EBUS probe was classified as within or adjacent to the PPL, as reported previously.[2] Post-TBB haemorrhage was defined as bleeding requiring further intervention or intensive care observation; self-limited bleeding was not counted as a complication. Post-biopsy pneumothorax was defined as accumulation of air in the pleural space, as confirmed by the chest x-ray. During the study period, chest x-ray was taken on an as-needed basis because of the favourable safety profile of EBUS-guided TBB.[15]

### Statistical analysis

Data were reported as No. (%) or mean±standard deviation, as appropriate. For comparisons between categorical variables, the χ^2^ or Fisher's exact test was used. With respect to the primary outcome, a multivariate logistic regression model was built to identify independent factors associated with a diagnostic EBUS-guided TBB. Variables with a p value of <0.2 were entered into the multivariate analysis. To investigate yearly trends in the diagnostic yield of TBB, we performed a linear-by-linear association χ^2^ test for categorical data. All analyses were conducted using SPSS for Windows, version 15.0 (SPSS Inc., Chicago, IL, US) and a 2-tailed p value of <0.05 was regarded as statistically significant.

## Results

### Patients

Over an 8-year period, a total of 2144 patients had been referred for EBUS-guided TBB of the PPLs, and 153 (7.1%) of them could not be localized by EBUS and EBUS-guided TBB was not attempted. The clinical characteristics of the study population are summarized in Table 1. The average patient age was 65±13 years (range, 20–100 years), and 1211 (57%) patients were male. The mean diameter of the PPLs was 3.4±1.8 cm. Localization of the PPLs was the upper lobes in half of the patients. The majority (87%) of the PPLs appeared solid on CT images and the final diagnosis was malignant in 1671 (78%) patients. The complication rate was low, with 1.8% and 0.5% for pneumothorax and haemorrhage, respectively. No procedure-related mortality was observed throughout the study period.

**Table 1:** Patient and clinical characteristics.

Characteristic	Value (n = 2144)
Age, years	65±13
Male sex	1211 (57)
Size of lesion	
≤ 2 cm	479 (22)
˃ 2 cm	1665 (78)
Lesion distribution	
Right upper lobe	569 (27)
Right middle lobe	320 (15)
Right lower lobe	392 (18)
Left upper lobe	500 (23)
Left lower lobe	363 (17)
Appearance on CT	
Solid	1856 (87)
Others^a^	288 (13)
Probe position	
Within	1423 (66)
Adjacent to or outside	721 (34)
Complication	
Pneumothorax	38 (1.8)
Haemorrhage	10 (0.5)
Final diagnosis	
Malignant	1671 (78)
Benign	473 (22)
Diagnostic yield of procedures	
Transbronchial biopsy	1502 (70)
Bronchial washing^b^	293 (17)
Bronchial brushing^c^	441 (40)

^a^ partly solid, pure ground-glass or cavitary.

^b^ 1747 patients undergoing bronchial washings were used as the denominator.

^c^ 1112 patients undergoing bronchial brushings were used as the denominator.

### Diagnosis

EBUS-guided TBB was diagnostic for 1547 (72%) of the PPLs. The diagnoses made by TBB are listed in Table 2. The diagnostic yield was associated with the size of the lesions, probe position and histologic nature of the PPLs (Table 3). In the multivariate analysis, patients with a lesion size of >2 cm, an EBUS probe placed within the PPL and a malignant lesion were more likely to have the diagnosis achieved by TBB (Table 4). For those non-diagnostic PPLs, the final diagnosis was achieved by CT-guided lung biopsy (n = 241), VATS biopsy (n = 234), biopsy from extra-pulmonary sites (n = 37), microbiology (n = 5), serology testing (n = 4), or clinical/radiological follow-up (n = 76).

**Table 2:** Diagnosis made by endobronchial ultrasound-guided transbronchial biopsy.

	No. (%) of patients
Diagnosis	(n = 1547)
**Malignant disease**	**1265 (82)**
Adenocarcinoma	850 (55)
Squamous cell carcinoma	144 (9.3)
Small cell carcinoma	70 (4.5)
Metastasis	67 (4.3)
Non-small cell carcinoma	65 (4.2)
Lymphoma	18 (1.2)
Carcinoma	18 (1.2)
Poorly differentiated carcinoma	12 (0.8)
Others	21 (1.4)
**Benign disease**	**282 (18)**
Tuberculosis	90 (5.8)
Chronic inflammation	67 (4.3)
Sarcoidosis	16 (1.0)
Cryptococcosis	14 (0.9)
Fibrosis	11 (0.7)
Organizing pneumonia	10 (0.6)
Pneumonia	8 (0.5)
Non-tuberculous mycobateriosis	7 (0.1)
Lung abscess	6 (0.4)
Pneumoconiosis	5 (0.3)
Hematoma	3 (0.2)
Mycetoma	2 (0.1)
Adenoma	2 (0.1)
Sclerosing haemangioma	1 (0.1)
Other benign process	40 (2.6)

**Table 3:** Patient and clinical features associated with diagnostic yield of endobronchial ultrasound-guided transbronchial biopsy.

Characteristic	Diagnostic yield by EBUS-guided TBB		p value
	No (n = 597)	Yes (n = 1547)	
Age, years			
≤ 65	297 (27)	803 (73)	0.370
˃ 65	300 (29)	744 (71)	
Gender			
Male	319 (26)	892 (74)	0.077
Female	278 (30)	655 (70)	
Size of lesion			
≤ 2 cm	212 (44)	267 (56)	<0.001
˃ 2 cm	385 (23)	1280 (77)	
Lesion distribution			
Upper lobes	285 (27)	784 (73)	0.222
Non-upper lobes	312 (29)	763 (71)	
Appearance on CT			
Solid	518 (28)	1338 (72)	0.866
Others	79 (27)	209 (73)	
Probe position			
Within	228 (16)	1195 (84)	<0.001
Adjacent to or outside	369 (51)	352 (49)	
Complication			
Pneumothorax			
Present	8 (21)	30 (79)	0.346
Absent	589 (28)	1517 (72)	
Haemorrhage			
Present	5 (50)	5 (50)	0.153
Absent	592 (28)	1542 (72)	
Final diagnosis			
Malignant	406 (24)	1265 (76)	<0.001
Benign	191 (40)	282 (60)	

EBUS, endobronchial ultrasound; TBB, transbronchial biopsy

**Table 4:** Multivariate logistic regression model for diagnostic yield of endobronchial ultrasound-guided transbronchial biopsy.

Characteristic		OR	(95% CI)	p value
Sex	Male vs. Female	1.23	(0.99–1.52)	0.054
Lesion size	˃ 2 cm vs. ≤ 2 cm	2.21	(1.75–2.78)	<0.001
Probe position	Within vs. Adjacent to or outside	5.11	(4.15–6.30)	<0.001
Complication	Haemorrhage vs. Non-haemorrhage	2.90	(0.78–10.78)	0.113
Final diagnosis	Malignant vs. Benign	1.97	(1.56–2.50)	<0.001

CI, confidence interval; OR, odds ratio

### Learning curves

**Fig 1:**
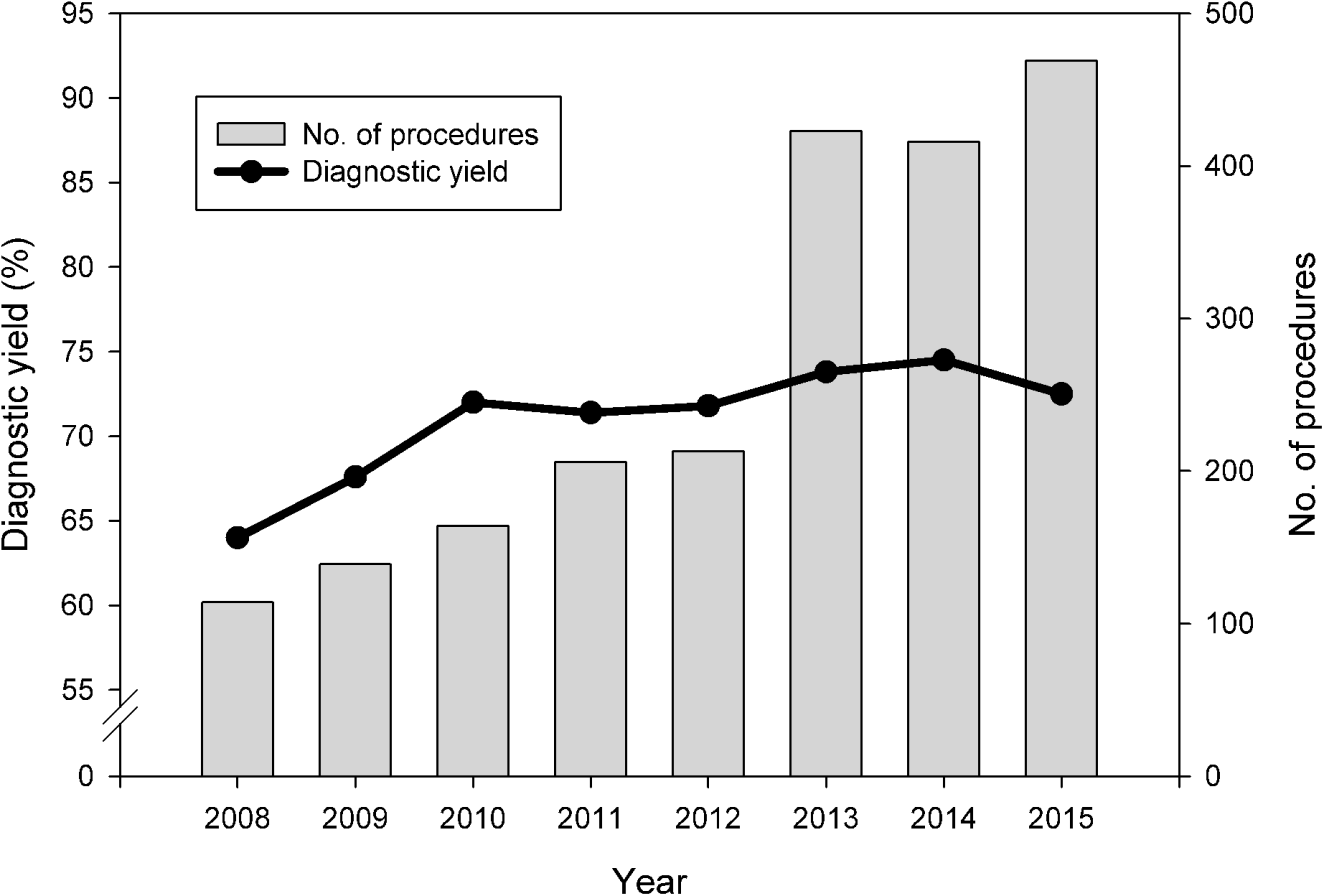
Diagnostic yield and case No. of endobronchial ultrasound-guided transbronchial biopsies over the study period.

The number of EBUS-guided TBB performed at our institution rose evidently between 2008 and 2015, and we observed a significant trend (p = 0.041) toward an increase in the diagnostic yield of TBB during the study period (Fig 1). The TBB yield increased from 64% in 2008 to reach a plateau of around 72% after 2010. In terms of the learning curve, approximately 400 EBUS-guided procedures should be conducted for a medical centre to obtain stable diagnostic proficiency.

**Fig 2:**
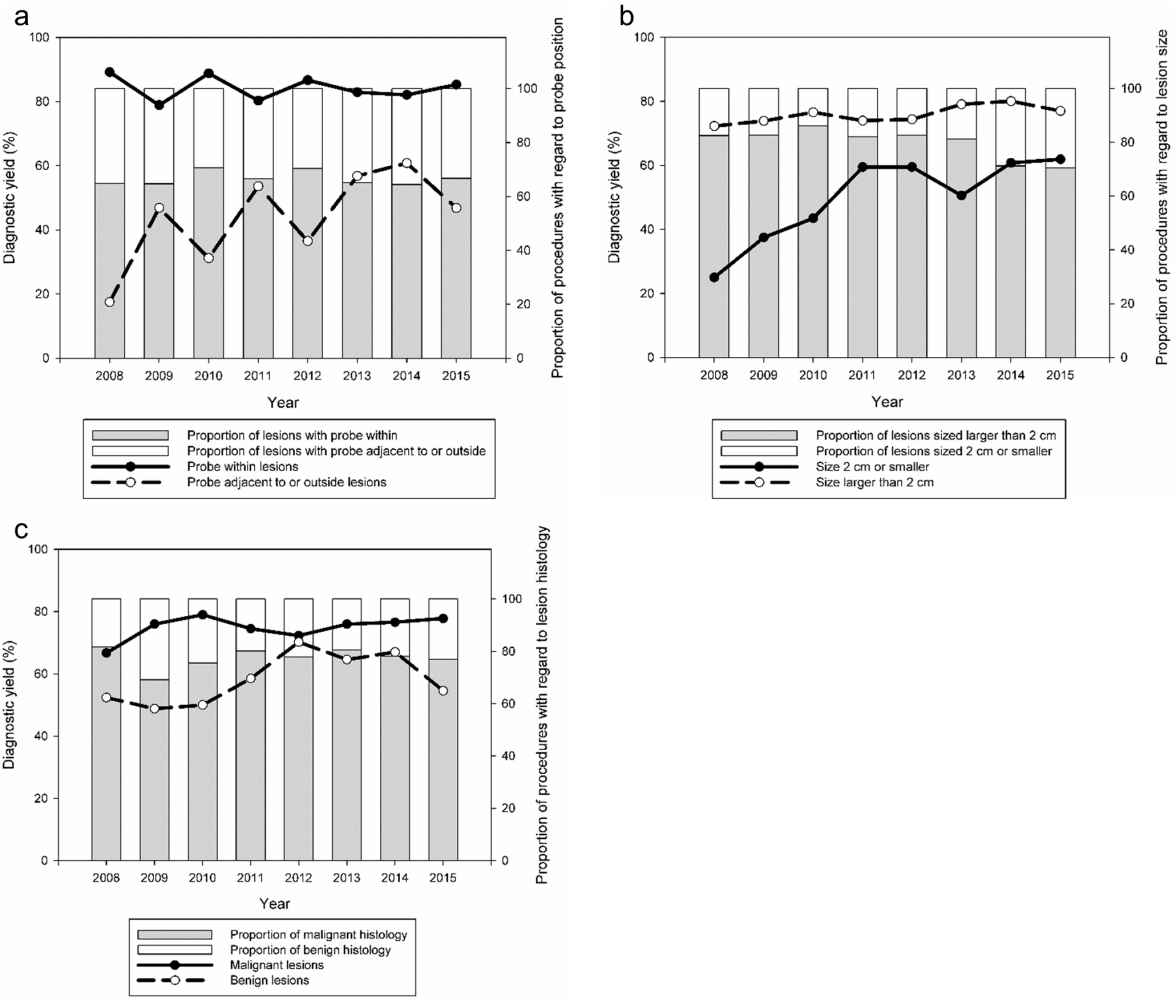
Diagnostic yield of endobronchial ultrasound-guided transbronchial biopsy and proportion of cases with regard to the probe position (a), lesion size (b) and histology (c).

Further in-depth analysis showed that the diagnostic performance of EBUS-guided TBB was similar with PPLs sized >2 cm, with the ultrasonic probe within, and with both benign and malignant histologies across the study years (Fig 2). A significant improvement in the diagnostic yield was observed in patients with the EBUS probe positioned adjacent to or outside the PPLs (Fig 2A) and in those with lesions with a size of ≤2 cm (p <0.001 and p = 0.001, respectively) during the study period (Fig 2B).

## Discussion

To our knowledge, this is the first study reporting on an institutional learning curve of EBUS-guided TBB in a large population and over a long period. The main findings of our study are as follows: (1) The diagnostic yield of EBUS-guided TBB for PPLs gradually improved over the first 3 years (or during approximately the first 400 procedures) and approached a plateau thereafter. (2) The improvement in TBB performance was mainly attributed to the increased TBB yield for PPLs with the probe adjacent to the lesions themselves and with an axial diameter of ≤2 cm. (3) The safety profile of EBUS-guided TBB for PPLs was excellent, as indicated by the extremely low risks of procedure-related complications. (4) The lesion size and histology, and ultrasonic probe position were significantly associated with the diagnostic yield of TBB.

Although EBUS-guided TBB is a relatively easy-to-learn task, as reflected by a nearly 70% diagnostic yield for PPLs in the first year of our practice and excellent initial results from other study groups,[1, 2] its performance could be refined over the subsequent 3 years or after hundreds of procedures. In our institution, the chest fellowship program runs for only 2 years; thus, the growth in experience of chest fellows may not fully explain the improvement in TBB yield. Instead, we speculate that maturation of the supervisors’ skill in EBUS-guided procedures and improvement in their tutoring practice contributed to the improved TBB performance over time. This highlights the importance of having experienced pulmonologists in EBUS-guided TBB services, in that their presence would be able to maintain stable proficiency at an institutional level and possibly foster the learning curve of their fellows.

An interesting and important finding in this study is that an advance in the diagnostic yield of TBB was observed only in patients in which the EBUS probe was adjacent to the PPLs and with lesions sized ≤2 cm. PPLs with these features were found to be associated with a lower diagnostic yield of EBUS-guided TBB in the present and prior studies;[2, 14, 16–18] thus, it may take time, experience and practice to achieve technical competence in dealing with these kinds of lesions. Similarly, the improved and steady diagnostic performance of EBUS-guided procedures for these PPLs observed in the later study years may be ascribed to the growing tutoring experience of the supervisor pulmonologists, who are able to help EBUS trainees do better early in their training course. On the other hand, we found a high and stable TBB yield in PPLs with a size of >2 cm, the EBUS probe within or malignant histology at the beginning of EBUS practice and throughout the study period, and this indicates a short learning curve for sampling such lesions. Taken together, it is suggested that we should be particularly dedicated to PPLs in which the measured diameter is ≤2 cm or the ultrasonic probe cannot be properly positioned within, in the EBUS training program for chest fellows.

In line with prior studies,[2, 14, 16, 18] the probe position relative to the PPL is the single most important determinant of the diagnostic yield of EBUS-guided TBB. The learner’s ability and the technique used to locate a PPL within the ultrasonic probe are prerequisites to becoming a qualified pulmonologist. Our results also support previous observations that the TBB yield is influenced by the PPL size.[17, 19, 20] The probability of malignant histology in PPLs is recognized to increase with an increasing lesion size;[21, 22] thus, some have proposed that this may explain the potential influence of lesion size on the performance of EBUS-guided TBB. However, even when lesion histology was taken into account in our study, lesion size still had an impact on the diagnostic yield of TBB. Moreover, comprehensive reviews and meta-analyses have found that the size of the PPL was a significant determining feature in TBB performance.[7, 8]

The overall diagnostic yield of EBUS-guided TBB in our institution falls within previously reported figures.[1, 16, 23–25] Steinfort *et al*.[7] recently demonstrated a clear positive correlation between the prevalence of malignancy in PPLs and diagnostic sensitivity of TBB, and our finding is close to the best-fitting straight line that they established. This suggests the comparability of our study results to those in previous reports.

Safety is an important issue for any kind of invasive procedures. For EBUS-guided TBB, pneumothorax and haemorrhage were the 2 most frequently encountered complications, in both our and other studies.[1, 2, 4, 16, 17, 24] Fortunately, the reported rates were low, ranging from 0–5.1%[2, 4, 16, 26] and 0–4.4%[2, 14, 16, 18, 26, 27] for pneumothorax and haemorrhage, respectively. Furthermore, there were no deaths in any study. The favourable safety profile renders this modality advantageous over CT-guided biopsy in the diagnosis of PPLs, although a superior diagnostic yield has usually been reported for the latter.[28] Nevertheless, pneumothorax and haemorrhage may complicate as much as 69%[29] and 10%[30] of CT-guided biopsy, respectively.

This study has several limitations. First, a learning curve for EBUS-guided TBB in a medical centre setting may not be entirely representative of a more general patient population in other hospital settings. However, we wanted to share our experience to increase understanding of such information and to encourage others to share theirs. Second, the EBUS-guided TBB procedure was accomplished by a cooperative team consisting of chest fellows with different levels of experience in our institution, and they were supervised by experienced pulmonologists. Thus, the impact of operator expertise on the institutional learning curve could not be accurately assessed and we did not describe individual learning curves in this study. What we have described can be considered a real-world scenario in a teaching hospital. Third, other ancillary bronchoscopic techniques, such as using a guide sheath, an ultrathin bronchoscope, fluoroscopy, virtual bronchoscopic navigation, electromagnetic navigation, and rapid on-site evaluation,[2, 20] were not available during the study period, so a direct comparison of data from other institutions applying these modalities and ours would be difficult. Nevertheless, this is also one of our study's strengths, in that it allows us a unique opportunity to observe the evolution of EBUS-guided TBB performance during a long-term period.

In conclusion, the EBUS-guided TBB may be an easy-to-learn technique, but it takes 3 years or around 400 procedures for a medical centre to achieve a better and more stable performance. In particular, the diagnostic yield of PPLs with a size of ≤2 cm and without the probe within could be clearly improved with time. Meanwhile, in this study, the largest patient population to date, we have demonstrated and confirmed that probe position, and lesion size and histology are significant determinants for a diagnostic TBB of PPLs. Finally, the most appealing feature of EBUS-guided TBB is its excellent safety profile, which makes it the preferred modality for sampling a PPL.

## Supporting information

S1 File Spreadsheet containing the dataset used in this study. (XLSX)
